# Recent findings on drug hypersensitivity in children

**DOI:** 10.3389/falgy.2024.1330517

**Published:** 2024-02-07

**Authors:** Mara Morelo Rocha Felix, Fábio Chigres Kuschnir, José Laerte Boechat, Mariana Castells

**Affiliations:** ^1^ Department of General Medicine, School of Medicine and Surgery, Universidade Federal do Estado do Rio de Janeiro, Rio de Janeiro, Brazil; ^2^ Department of Pediatrics, Faculty of Medicine, Universidade do Estado do Rio de Janeiro, Rio de Janeiro, Brazil; ^3^ Clinical Immunology Service, Internal Medicine Department, Faculty of Medicine, Universidade Federal Fluminense, Niterói, Brazil; ^4^ Basic and Clinical Immunology Unit, Department of Pathology, Faculty of Medicine, University of Porto, Porto, Portugal; ^5^ Division of Rheumatology, Allergy and Immunology, Department of Medicine, Brigham and Women’s Hospital, Boston, MA, United States

**Keywords:** drug hypersensitivity, children, allergy, antibiotic, vaccines, severe cutaneous adverse drug reactions, Stevens-Johnson syndrome, toxic epidermal necrolysis

## Abstract

Drug hypersensitivity reactions (DHR) in children have a significant impact on clinical practice and public health. Both under-diagnosis (due to under-reporting) and over-diagnosis (due to the overuse of the term “allergy”) are potential issues. The aim of this narrative review is to describe the most recent findings of DHR in children/adolescents and gaps regarding epidemiology, antibiotic allergy, antiepileptic hypersensitivity, vaccine allergy, and severe cutaneous adverse reactions (SCAR) in this age group.

## Introduction

Drug hypersensitivity reactions (DHR) in children/adolescents have a significant impact on clinical practice and public health (1, 2). Both under-diagnosis (due to under-reporting) and over-diagnosis (due to the overuse of the term “allergy”) are potential issues. Antibiotic allergy is often reported and not properly diagnosed in pediatric age groups (1, 2). Maculopapular exanthems (MPE) and even urticaria during treatment with antibiotics, especially beta-lactams (BL), are often labeled as drug allergy, but the vast majority are caused by infection or the interaction between medication and viruses (1, 2). Furthermore, other adverse drug reactions (ADR), such as headache or gastrointestinal intolerance, are mistaken for real allergic reactions (1, 2).

DHR are unpredictable reactions that can be reproduced by administering a suspected drug at doses normally tolerated by other individuals (3–5). They may be mediated by immunological (allergic types) or non-immune (non-allergic types) mechanisms (4, 5). DHR can also be classified according to the time they occur after drug administration (4, 5). Immediate reactions may occur within 1–6 h after the first dose of the drug and are generally IgE-mediated (4–6). Non-immediate reactions occur after 1 h, but more frequently after days of drug administration, and are generally mediated by *T* cells (4–6).

The aim of this article is to describe the most recent findings of DHR in children/adolescents and gaps regarding epidemiology, antibiotic allergy, antiepileptic hypersensitivity, vaccine allergy, and severe cutaneous adverse reactions (SCAR) in this age group.

## Methods

To write this narrative review, the authors searched for information on PubMed and selected publications from January 1990 to July 2023, using the following keywords: “drug hypersensitivity”; “antibiotic allergy”; “antiepileptic hypersensitivity”; “vaccine”; “severe cutaneous adverse reactions”; “childhood”; “pediatric”; and “adolescence”. The inclusion criteria were any type of publication (systematic reviews, randomized clinical trials, observational studies, case series, position statements, and selected reviews) on drug hypersensitivity related to children and adolescents, written in English.

The selection of topics considered the drugs most involved in DHR in children, the most severe reactions, and some issues of special interest in the pediatric age group, such as allergy to vaccines and antiepileptic drugs. For articles, the selection was made according to relevance, date of publication, and inclusion of pediatric patients. Therefore, in general, more recent articles and some reviews focused on pediatric age published by recognized researchers in drug allergy were included. Being a mini review, a limitation of this article is that it could not cover all topics related to pediatric drug allergy.

## Epidemiology of drug allergy in children

There are fewer epidemiological studies on drug allergy in children compared to adults. Most of them reveal that many children with suspected drug allergies are not, in fact, allergic (7, 8). Unfortunately, they often carry this false allergy label into adulthood (7, 8). It is important to highlight that the diagnosis of DHR should be based not only on clinical history, but also on skin tests (ST), validated *in vitro* tests, and drug provocation tests (DPT) (6, 8).

Recently, Capanoglu et al. evaluated 5,553 children with suspected drug allergies (9). Parents were asked “Has your child ever developed any allergies after using drugs?”, and 7% (389) answered that their child had drug allergies. Pediatric allergists suspected that 21.1% (*n* = 82/389) had drug allergies. After diagnostic tests, drug allergy was confirmed in 4.2% (*n* = 3/72). Therefore, the frequency of suspected drug allergy was 1.47% (*n* = 82/5553), and the prevalence of confirmed drug allergy was 0.05% (*n* = 3/5,553) (9).

Another study investigating DHR in children was conducted in Serbia (10). They evaluated 504 patients, with a mean age of 7.5 years. There were 375 children with a history suggestive of single-drug hypersensitivity and 129 with multiple-drug hypersensitivity. The main drugs involved were antibiotics (83%), non-steroidal anti-inflammatory drugs (NSAIDs) (8.4%), and antipyretics (3.8%). There was skin involvement in 96.2%. DHR were confirmed in 4.4% —six patients had positive ST and 13 had a positive DPT. In the proven DHR group, the main culprits were antibiotics (72.7%), followed by NSAIDs (8.3%). Urticaria was the most common skin manifestation (78.2%), followed by exanthema (10.5%) and angioedema (5.3%). The presence of extracutaneous manifestations was significantly associated with a positive allergy test (*p* = 0.022, OR 4.62, 95% CI 1.05–15.76%) (10).

## Antibiotic allergy and its impact on child health

The most frequent cause of DHR mediated by immune mechanisms in children is antibiotics, especially BL (11). Around 10% of parents report an allergy to BL in their children (12). After proper evaluation by an allergist, nearly 95% of these children will be able to tolerate the antibiotic (8, 11, 12). In this age group, infections are more common (mainly viral infections) and can mimic drug allergies or act as cofactors.

The “penicillin allergy” label is associated with many adverse effects in individual and global health. The patient labeled as “allergic to penicillin” uses more broad-spectrum antibiotics, such as fluoroquinolones, vancomycin, and clindamycin, increasing the prevalence of infections by bacteria such as *Clostridium difficile*, methicillin-resistant *Staphylococcus aureus*, and vancomycin-resistant *Enterococcus* (13–15). In addition, studies have shown that these patients are at greater risk of postoperative complications, longer hospital stays, greater cost of treatment, and higher rate of treatment failure (13–15).

Taylor et al. investigated the epidemiology and factors associated with penicillin allergy labels in two large US pediatric primary care networks (16). They conducted a retrospective, longitudinal birth cohort study in 90 pediatric primary care clinics with 334,465 children born between January 2010 and June 2020. They found that children were labeled early in life (mean age 1.3 years), and nearly half were labeled after receiving 1 or 0 penicillin prescriptions. These findings are alarming and question the validity of penicillin allergy labels (16).

Another study demonstrated the impact of an antibiotic allergy label in childhood. Lucas et al. evaluated 1,672 pediatric patients from Perth, Australia, and found antibiotic allergy labels in 5.3% of patients, most of them BL allergy labels (85%) (17). The incidence of antibiotic allergy labels increased with age (*p* < .001). Patients with antibiotic allergy labels received more macrolide, quinolones, lincosamide, and metronidazole antibiotics than patients without an antibiotic allergy label (17). Furthermore, children with any antibiotic or BL allergy label had longer hospital stays (odds ratio, 1.62; 95% CI, 1.05–2.50), with a mean length of hospital stay of 3.8 days for those without a label and 5.2 days for those with a BL allergy label (17).

Appropriate assessment of antibiotic allergy is an essential part of antibiotic stewardship program efforts. Removing false labels from a patient with suspected drug allergies may reduce unnecessary exclusions. Thus, there are several publications aiming to optimize the investigation of allergy to BL (18–20). Recently, an EAACI task force published a review on BL allergy in children (21). In this article, the authors stated that DPT is almost universally advocated in non-immediate mild MPE, with sufficient evidence that ST is not mandatory in this situation (21). However, the index reaction history must be reliable, showing low risk for the patient. The ideal protocol for DPT remains to be defined. For mild immediate BL reactions, further studies are needed to confirm the safety of performing a direct DPT in children (21).

Finally, in 2023, a systematic review investigated the safety of performing direct DPT with BL without ST to guide the decision for re-exposure in children with mild BL reactions (22). They found a low prevalence of BL reactions by direct DPT (5.23%) and a very low frequency of severe reactions from direct DPT (0.036%), supporting direct DPT as a safe and effective delabeling tool in children with suspected mild BL reactions (22).

## Antiepileptic hypersensitivity reactions

Antiepileptic drugs (AED) are often prescribed in childhood and are one of the most common causes of DHR in this age group (23). These reactions may present with benign MPE, but severe cutaneous adverse reactions (SCAR) are sometimes observed, such as Stevens-Johnson syndrome (SJS), toxic epidermal necrolysis (TEN), and drug reaction with eosinophilia and systemic symptoms (DRESS) (23). Acute generalized exanthematous pustulosis (AGEP) is also a SCAR, rarely caused by AED (23).

AED are classified as aromatic or non-aromatic depending on the presence of at least one aromatic ring (23). Aromatic AED (phenobarbital, phenytoin, carbamazepine, oxcarbazepine, lamotrigine, felbamate, zonisamide, and primidone) are responsible for the most serious reactions (23). In a Turkish study that evaluated 58 pediatric patients with SCAR, antibiotics were the most common culprit medication (51.7%) and AED were the second most common (31%) (24). Another analysis of two multicenter case-control studies showed that anti-infective sulfonamides and AED, especially phenobarbital, carbamazepine, and lamotrigine, were the drugs most associated with the risk of SJS/TEN in children (25). Levetiracetam is a new non-aromatic AED with a better tolerability profile. However, there are also some reports of severe reactions to this drug (23).

There are several factors involved in the pathogenesis of these DHR. Most of them are non-immediate type IV reactions, explained by the hapten/prohapten, p-i concept, and altered peptide repertoire hypotheses (23). However, other theories are being proposed to help understand the mechanisms related to these AED hypersensitivity reactions (AED-HR). The “danger hypothesis” postulates that some signals derived from viral infections or drug metabolism may act as cofactors to promote immune modulation (23). Furthermore, genetic polymorphisms and HLA alleles have been described as risk factors for certain SCAR in specific populations (23). The associations between HLA alleles/genetic polymorphisms and AED-HR observed in studies with children are presented in Table 1 (26–30).

**Table 1:** Associations between HLA alleles/genetic polymorphisms and AED-HR among children.

HLA allele	AED-HR	Population	Study
HLA-A*31:01	Carbamazepine-induced DRESS and MPE	North America children	Amstutz U et al. (26)
HLA-B*15:02	Carbamazepine-induced SJS	North America children	Amstutz U et al. (26)
HLA-B*15:02	Carbamazepine-induced SCAR	Singapore children of Chinese and Malay ethnicity	Chong KW et al. (27)
HLA-A*01:01 and HLA-B*13:01	Phenobarbital hypersensitivity	Thai children	Manuyakorn W et al. (28)
CYP2C9*3 variant	Phenytoin-induced SCAR	Thai children	Suvichapanich S et al. (29)
CYP2C19*2 variant	Phenobarbital-induced SCAR	Thai children	Manuyakorn W et al. (30)

AED-HR, hypersensitivity reactions caused by antiepileptic drugs; DRESS, drug reaction with eosinophilia and systemic symptoms; MPE, maculopapular exanthema; SCAR, severe cutaneous adverse reactions; SJS, Stevens-Johnson syndrome.

Management of patients with a history of AED hypersensitivity is difficult, as many of these patients require ongoing treatment of seizures. Avoidance of all aromatic AED is generally recommended in patients who react to one of these medications, and non-aromatic AED may be an option (23).

## Vaccine allergy

Vaccines and their components can cause hypersensitivity reactions. However, true hypersensitivity to vaccines is rare and signs and symptoms appearing after vaccination may be coincidental (31, 32). Vaccination is a public health action, and it is necessary to evaluate children through a complete allergy work-up. Vaccine hesitancy is one of the biggest threats to global health, emphasizing the importance of allergists and pediatricians in promoting vaccine safety (31, 32).

True vaccine allergy is found in <10% of children investigated in allergy units after a potential vaccine hypersensitivity reaction (31). A vaccination reaction should be referred to as an adverse event following immunization (AEFI) until it can be categorized (31).

All vaccines can cause immediate and non-immediate immunological reactions. Fortunately, most of these reactions are mild. Anaphylaxis and SCAR are extremely rare (32, 33). When investigating patients with AEFI, it is important to consider that individual vaccine components, including active immunizing antigens, conjugating agents, preservatives, stabilizers, antimicrobial agents, adjuvants, and culture media, may be possible allergic triggers (31, 32). Specific guidelines including testing with the different vaccine components as well as the vaccine itself have been published (33–35).

Fear of an allergic reaction can cause vaccine hesitancy. This has recently been observed with COVID-19 vaccines, which have been associated with cutaneous and systemic reactions. However, the incidence of anaphylaxis after COVID-19 vaccines is comparable to that of other vaccines (36). Another rare AEFI found in children and adolescents, especially in male adolescents after receiving mRNA vaccines, is myocarditis/pericarditis (37). These patients require close monitoring, but most of them improve quickly after treatment (37). Considering the risk/benefit ratio, an international guideline published in 2022 suggested vaccination against COVID-19 for children/adolescents aged between 3 and 17 years, monitoring possible side effects after vaccination (38).

One of the most common situations involving vaccine allergies is the evaluation of children with suspected food allergy (e.g., egg, milk, gelatin) (34). Regarding egg-allergic children, several studies have already demonstrated the safety of the measles-mumps-rubella (MMR) vaccine for these patients (34). This is also true for most injected inactivated influenza vaccines (34). The management of egg-allergic patients who need a yellow fever vaccine (YFV) is more complex. A Brazilian study investigated the safety of YFV in confirmed egg-allergic patients and concluded that the administration of YFV using a specific protocol was safe in these patients (39). The IDT was useful in predicting a higher risk of vaccine reaction (39).

In children with systemic and/or cutaneous mastocytosis, vaccines may trigger hypersensitivity reactions by mast cell mediator release. Some experts recommend premedication after a reaction in these patients; however, there is no consensus regarding this issue (40). Avoiding the simultaneous administration of multiple vaccines is another measure that may be useful (40).

## Severe cutaneous adverse reactions (SCAR) in children

Severe cutaneous adverse drug reactions are rare conditions that can be fatal. They include DRESS, SJS/TEN, and AGEP.

A Turkish study mentioned above investigated pediatric patients with SCAR and found a median age of 8.2 years (50% were male) (24). There was SJS/TEN in 60.4% (*n* = 35), DRESS in 27.6% (*n* = 16), and AGEP in 12% (*n* = 7) of patients (24). Drugs were the cause of reactions in 93.1% of children, mainly antibiotics and AED. Only one patient (with TEN) died (24). Another study conducted in France analyzed 49 pediatric cases of DRESS (41). The median age was 8 years (44.9% were male). The most frequent culprit drugs were antibiotics (65%) and AED (27.5%) (41).

### Drug reaction with eosinophilia and systemic symptoms (DRESS)

DRESS is a severe and potentially fatal drug-induced, cell-mediated reaction (41.42). AED (mainly aromatic) and antibiotics are the most commonly involved medications (41, 42). The incidence of DRESS due to anticonvulsants is 1:1,000–1:10,000 in the general population (43) and 0.4:1,000 (44) in hospital settings. Its incidence is lower in children than in adults (42), and the mortality rate is approximately 10%, with a lower percentage in children than in adults (42, 45).

The exact pathogenic mechanism of DRESS is unknown, but some genetic studies have found an association between HLA haplotypes and susceptibility to DRESS (26, 30). Furthermore, there appears to be a role for viral reactivation during DRESS, especially Human Herpes virus (HHV-6), as well as Epstein Barr virus (EBV), cytomegalovirus (CMV), and HHV-7 (46).

The time latency of DRESS symptoms ranges from 2 to 6 weeks after initiation of treatment. In children, the average age of occurrence of DRESS syndrome is 9 years of age (42, 45). The main clinical manifestations of DRESS are fever (usually high); facial edema; MPE (but other cutaneous reactions may occur, such as urticarial, pustular, bullous, lichenoid, exfoliative, and eczematous rashes); lymphadenopathy; hematological abnormalities, like leukocytosis with eosinophilia and/or atypical lymphocytosis; and organ involvement (hepatitis, nephritis, pneumonitis) (41, 42).

The diagnosis of DRESS is based on different criteria. The most frequently used are the European Registry of Serious Cutaneous Adverse Drug Reactions (RegiSCAR) or the Japanese Serious Cutaneous Adverse Drug Reactions (SCAR-J) group (47, 48). They include clinical signs and symptoms (fever, rash), hematological abnormalities, and organ involvement (47, 48). The Japanese group also included HHV-6 reactivation in their criteria (48).

Treatment begins with the withdrawal of all medications suspected of causing DRESS (41, 42). Mild cases are treated with topical corticosteroids and emollients. Moderate and severe cases are usually treated with systemic corticosteroids (41, 42). There are case reports of good response to other medications, including cyclosporine, azathioprine, infliximab, and mycophenolate, in patients with more severe disease (42). Intravenous immunoglobulin (IVIG) may be useful in some patients, but its benefit is still controversial (42).

### Stevens-Johnson syndrome (SJS) and toxic epidermal necrolysis (TEN)

SJS and TEN are rare severe cutaneous adverse drug reactions mediated by cytotoxic *T* cells (mainly CD8+). There is extensive epidermal necrosis and detachment, with mucocutaneous complications (49, 50). SJS and TEN differ only along a spectrum of severity based on percentage of body surface area detached (<10% in SJS; 10%–30% in an overlap SJS/TEN; and >30% in TEN) (49). Hsu et al. reported an incidence rate of 5.3 and 0.4 cases per million children for SJS and TEN, respectively, with an equal incidence between male and female children (51). In children, as in adults, drugs are the most frequent triggers of SJS/TEN (49, 50). Common culprit medications are sulfa antibiotics, AED, and NSAIDs (49).

The early manifestations of SJS/TEN include fever, malaise, and anorexia (50). It progresses with cutaneous lesions and bullae. SJS/TEN can mimic other illnesses, such as erythema multiforme major (EMM), staphylococcal scalded skin syndrome (SSSS), or autoimmune blistering diseases (49, 50). Table 2 shows some differences in clinical characteristics and main causes between SJS/TEN and other cutaneous diseases (49–56).

**Table 2:** Differential diagnosis of SJS/TEN and other cutaneous disorders in children.

Diagnosis	Clinical manifestations	Main causes
SCAR		
SJS/TEN	Prodromal symptoms (high fever, headache, malaise). Prominent involvement of mucosal surfaces (at least 2 sites). Epidermal detachment with Nikolsky's sign of the bullae (sloughing of the superficial skin layer with slight rubbing pressure). Lesions manifest first on the face and thorax and then spread outward symmetrically. Distal portions of the arms and legs are relatively spared but palms and soles can be an early site for lesions. Atypical “target” lesions (two concentric zones of color change).	Antiepileptics, antibacterial sulfonamides, nevirapine, NSAIDs, antituberculosis drugs
DRESS	Erythematous urticaria-like or violaceous skin eruption, facial and extremity edema, lymphadenopathy, fever, internal organ involvement, and laboratory abnormalities (increased concentrations of creatinine and liver enzymes; hematological abnormalities such as eosinophilia or lymphocytosis).	Antiepileptics, antibiotics (sulfonamides, vancomycin)
AGEP	Non-follicular sterile pustular rash over widespread erythema, fever, and neutrophilia.	Antibiotics (penicillins, cephalosporins), antimycotics, analgesics
Infectious		
SSSS	Prodromal manifestations (sore throat, purulent conjunctivitis, fever, malaise). Generalized erythema may progress to blisters and scaling, but the lesions are never dark or purpuric. Blisters often affect the flexures. There is no involvement of the mucous membrane. Nikolsky's sign is present.	*Staphylococcus* exfoliative exotoxins A and B.
EMM	Benign cutaneous illness with or without mucous membrane involvement (usually limited to the oral cavity). Minimal constitutional symptoms. Symmetric erythematous eruptions on the dorsum of the hands and the feet and on the extensor surfaces of arms and legs; palms and soles are usually involved. Truncal involvement is sparse. Face and scalp are rarely involved. Involvement of less than 10% of the body surface area. “Target” or “iris” lesions are pathognomonic. Typical target lesions are individual lesions less than 3 cm in diameter with a regular round shape, well-defined border, and at least three different zones, i.e., two concentric rings around a central disk. A ring consists of palpable edema, paler than the central disk.	The causes of EM are mostly viral (80% to 90%) or drug-related. Infections: HSV-1 (most common cause), HSV-2, CMV, EBV, influenza, and COVID-19. Drugs: Antibiotics (e.g., erythromycin, nitrofurantoin, penicillins, sulfonamides), antiepileptics, NSAIDs, and vaccines. Other conditions: IBD, hepatitis C, leukemia, lymphoma, and solid-organ cancer malignancy.
*Mycoplasma pneumoniae*-associated mucocutaneous disease	Eruptions include mucositis alone, prominent mucositis with sparse cutaneous involvement, and mucositis with moderate skin involvement. Lesions are characterized as vesiculobullous, targetoid, atypical targets, or macules.	*Mycoplasma pneumoniae*
Autoimmune		
Bullous dermatosis of childhood (linear IgA bullous dermatosis)	Annular and arcuate groups of tense bullae encircling the central crust. This morphology is referred to as a “crown of jewels” or “string of pearls”. The most affected areas are the face, lower part of the trunk, groin, and extremities (possible mucosal involvement).	IgA autoantibody against a component of bullous pemphigoid antigen (BPAG) 180
Bullous pemphigoid	Scattered tense bullae. In infants, palmoplantar involvement is more common, while groin involvement is more common in older children.	Autoantibodies to dermal-epidermal adhesion molecules (BPAG 180 and BPAG 230)
Bullous lupus erythematosus	Localized or generalized vesicles and bullae arising on erythematous or normal-appearing skin.	Autoantibodies to collagen VII

AGEP, acute generalized exanthematous pustulosis; BPAG, bullous pemphigoid antigen; CMV, cytomegalovirus; DRESS, drug reaction with eosinophilia and systemic symptoms; EBV, Epstein-Barr virus; EMM, erythema multiforme major; HSV-1, Herpes Simplex virus type 1; HSV-2, Herpes Simplex virus type 2; IBD, inflammatory bowel disease; NSAIDs, non-steroidal anti-inflammatory drugs; SCAR, severe cutaneous adverse reactions; SSSS, staphylococcal scalded skin syndrome; SJS, Stevens-Johnson syndrome; TEN, toxic epidermal necrolysis.

In SJS/TEN, the denuded skin areas may be associated with pain, fluid and protein loss, electrolyte imbalances, and bleeding (49). They also predispose to bacterial superinfection, most commonly by *Staphylococcus aureus* and *Pseudomonas aeruginosa* (49). Ophthalmic disorders with sequelae are frequent, affecting up to 30% of children and adults (49).

The treatment is based on early detection and removal of the offending agents. Patients should be admitted to an intensive care unit or burn center as quickly as possible (49, 50). Supportive care is comprised of wound and eye care; fluid and electrolyte management; nutritional support; and prompt treatment of infections (49, 50). Systemic treatment includes corticosteroids, cyclosporine, and etanercept, with some promising outcomes (50). IVIG in high doses (2 g/kg) is another therapeutic option (49, 50).

### Acute generalized exanthematous pustulosis (AGEP)

AGEP is a rare, severe cutaneous adverse reaction. It is most commonly caused by drugs but can also be triggered by infections, especially in children (52). It is characterized by fever, pinhead-sized nonfollicular sterile pustules on erythematous skin lesions mostly in the face and intertriginous areas, leukocytosis, and rare organ involvement (52). Among all the SCAR, it is considered less severe with a lower rate of mortality (52).

## Conclusions

Drug allergy in children is an important issue, with an impact on individual healthcare, limiting treatment options and causing more adverse effects, and on public healthcare, increasing bacterial resistance, costs, and length of hospital stay. Many of these young patients carry allergy medication labels into adulthood. Therefore, the removal of false allergy labels in this age group should be encouraged as part of public health policies. Strategies to optimize BL allergy investigation, such as direct DPT without ST, are being implemented. Studies investigating the most frequent DHR, optimized diagnostic methods, and more appropriate management in pediatrics should be encouraged.
